# A learning health systems approach to improving the quality of care for patients in South Asia

**DOI:** 10.1080/16549716.2019.1587893

**Published:** 2019-04-05

**Authors:** A. Beane, D. Wagstaff, A. Abayadeera, M. Wijeyaratne, G. Ranasinghe, S. Mirando, A. M. Dondorp, D. Walker, R. Haniffa

**Affiliations:** a Department of Malaria, Mahidol Oxford Tropical Research Unit, Bangkok, Thailand; b University of Amsterdam, Netherlands; c Network for Improving Critical Care Systems and Training, Colombo, Sri Lanka; d PQIP, National Institute of Academic Anaesthesia Health Services Research Centre, Royal College of Anaesthetists, London, UK; e Surgical Outcomes Research Centre, University College London, London, UK; f Department of Surgery, Faculty of Medicine, University of Colombo, Colombo, Sri Lanka; g Department of Surgery, Faculty of Medicine, University of Colombo, Colombo, Sri Lanka; h Institute of Cardiologist, National Hospital Sri Lanka, Colombo, Sri Lanka; i Centre for Perioperative Medicine, Division of Surgery and Interventional Science, University College London, London, UK; j Anaesthesia and Critical Care Medicine, University College London Hospitals, London, UK; k Bloomsbury Institute for Intensive Care Medicine, Division of Medicine, University College London, London, UK

**Keywords:** Quality improvement, acute care, capacity building, surveillance, learning health systems

## Abstract

Poor quality of care is a leading cause of excess morbidity and mortality in low- and middle- income countries (LMICs). Improving the quality of healthcare is complex, and requires an interdisciplinary team equipped with the skills to design, implement and analyse setting-relevant improvement interventions. Such capacity is limited in many LMICs. However, training for healthcare workers in quality improvement (QI) methodology without buy-in from multidisciplinary stakeholders and without identifying setting-specific priorities is unlikely to be successful. The Care Quality Improvement Network (CQIN) was established between Network for Improving Critical care Systems and Training (NICST) and University College London Centre for Perioperative Medicine, with the aim of building capacity for research and QI. A two-day international workshop, in collaboration with the College of Surgeons of Sri Lanka, was conducted to address the above deficits. Innovatively, the CQIN adopts a learning health systems (LHS) approach to improving care by leveraging information captured through the NICST electronic multi-centre acute and critical care surveillance platform. Fifty-two delegates from across the CQIN representing clinical, civic and academic healthcare stakeholders from six countries attended the workshop. Mapping of care processes enabled identification of barriers and drivers to the delivery of care and facilitated the selection of feasible QI methods and matrices. Six projects, reflecting key priorities for improving the delivery of acute care in Asia, were collaboratively developed: improving assessment of postoperative pain; optimising sedation in critical care; refining referral of deteriorating patients; reducing surgical site infection after caesarean section; reducing surgical site infection after elective general surgery; and improving provision of timely electrocardiogram recording for patients presenting with signs of acute myocardial infarction. Future project implementation and evaluation will be supported with resources and expertise from the CQIN partners. This LHS approach to building capacity for QI may be of interest to others seeing to improve care in LMICs.

## Background

Addressing deficiencies and inequities in acute and critical care in resource-poor settings remain global health priorities [1–4]. Poor quality of care has been identified as a leading cause of excess mortality and morbidity in low- and middle-income countries (LMICs); most notably in South Asia, where poor quality exceeds limited access to or unavailability of care as a cause of mortality [3,5]. In response, the Lancet Global Health Commission on Care Quality has set out four key actions to raise the quality of healthcare delivered: building a shared vision of care quality, a clear strategy for quality evaluation, stronger regulation ensuring civic and professional accountability, and continuous learning [5].

Capacity for capturing information to evaluate the quality of care and measure the impact of interventions in LMICs is limited [6,7]. Internationally, the information needed to evaluate and benchmark healthcare is increasingly derived from electronic surveillance systems, implemented at facility level and scaled nationally [8,9]. Such systems are only now starting to emerge in LMICs where local collaborators emphasise that continuous reporting of processes of care, essential for evaluating quality, is often too burdensome [10–12]. In addition, health system evaluation and quality improvement methods are neither an established part of medical education nor prioritised by clinicians faced with daily assault of delivering frontline care with limited resources [13]. Subsequently, quality improvement (QI) initiatives in LMICs are often led by external experts, who may have limited insight into determinants of care, or of discreet organisational or cultural barriers to implementation of QI initiatives [2,6]. While the importance of north–south partnerships to facilitate transfer of such skills is being increasingly recognised, practical initiatives to engage interprofessional teams remain uncommon [10].

### Care Quality Improvement Network

The Care Quality Improvement Network (CQIN) [11] is a collaboration between University College London Centre for Perioperative Medicine (UCL-CPOM) [14] and Network for Improving Critical care Systems and Training (NICST) [12]. NICST is a non-profit organisation which has developed a clinician-led, setting-adapted continuous surveillance platform, supporting the delivery of acute medical, surgical and critical care for over 250,000 patients in South Asia [11,15,16]. The platform, part of a learning health system (LHS) methodology to improve acute care in LMICs, has enabled evaluation of patient outcomes, development of prognostic models, observational research and quality improvement through real-time information feedback and clinical training [11,15–17]. The CQIN aims to develop an international network of health care workers, researchers, educators and administrators with capacity to improve acute care in LMIC settings.

## Aim

This short communication describes an innovative learning health systems approach to identifying setting-relevant priorities for improving the quality of care using routine clinical data captured through digital health information platforms.

## Approach

Using a health systems approach, structured discussions with frontline clinicians and stakeholders examined existing information from three NICST registries (cardiology, critical care, surgery) to select candidate QI themes [13,18]. The themes selected were: internationally recognised indicators of quality of the processes and outcomes of care; routinely used tools for assessment of risk, complication and recovery; and efficiency of treatment pathways [19,20]. Examples include the time between referral and an intervention, patient satisfaction and complications following intervention. These replicable, objective processes, common to the cardiology, critical care and surgical care patient pathways, were chosen to provide a focus through which groups at the workshop could identify clear gaps in existing care that may be amenable to quality improvement.

Interprofessional stakeholders from the existing NICST registry partnerships and participants in UCL-CPOM’s academic programmes were invited to attend a two-day workshop in Sri Lanka. Healthcare researchers with expertise in health systems and quality improvement, alongside experienced clinicians who have undertaken change and improvement in their clinical settings, were recruited as faculty. The workshop was affiliated with the academic sessions of the College of Surgeons of Sri Lanka, themed as ‘*Striding towards equity and excellence in surgical care*’. During the workshop, measures of processes and outcomes of care, accessible through online live dashboards, were used to facilitate data-driven conversations regarding existing quality of care [13]. CQIN faculty with expertise in QI, health informatics and health systems research, all with experience in South Asia, supported the delegates with the evaluation. Workshop sessions focused on the definition, measurement and evaluation of quality. Care as expected for the surgical, acute myocardical infarction (AMI) and critical care patients was analysed using process mapping tools available through the Institute for Health Improvement (IHI). Actual care was then described by the delegates, informed by the digital registries.

Digital analytics dashboards (accessible as part of the NICST collaborative platform) displaying process and outcome measures were used to facilitate data-driven conversations regarding existing delivery of care from three acute-care specialities (cardiology, critical care, surgery) [21]. CQIN statisticians, clinicians, health informatics researchers and behavioural scientists, all with experience in South Asia, supported the delegates with interpretation of the information. Workshop sessions focused on the definition, measurement and feedback of quality. Driver diagrams and facilitated discussion with the delegate groups were used to elicit enablers and potential barriers to care.

The CQIN was registered with the IHI, enabling access to the IHI’s online resources during the workshop [23]. The workshop culminated in presentation of the project proposals and led to discussions on practical steps aiding implementation. Project proposals for the QI initiatives and ethics applications (if needed) were developed and delegate feedback captured.

## Priorities for improvement

**Figure 1.**
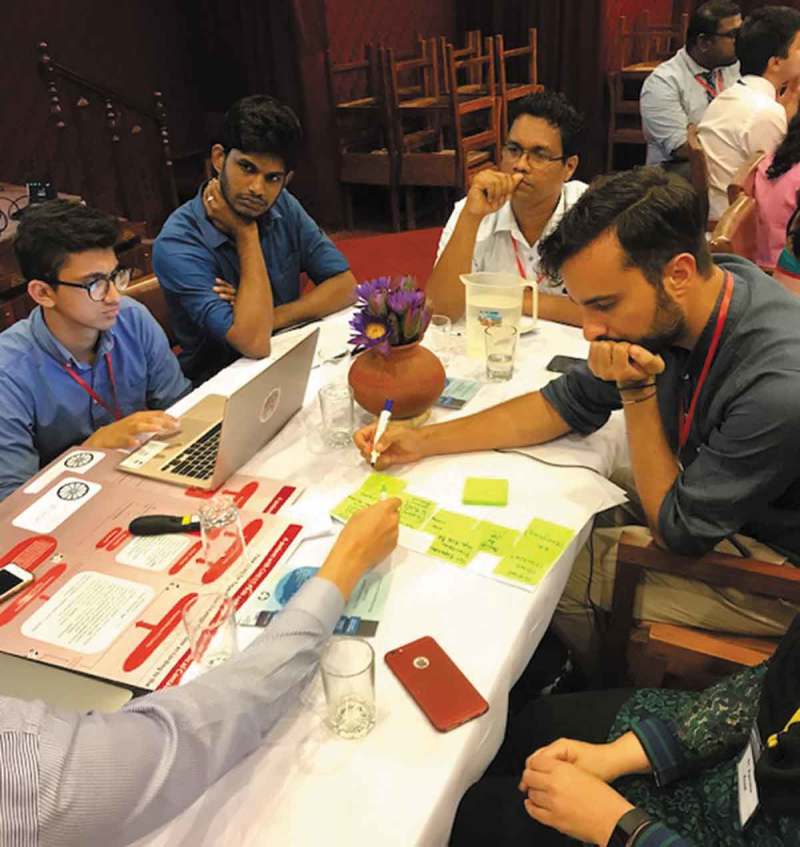
Participants mapping care as expected for acute care using process mapping tools, facilitated by international faculty.

Fifty-two delegates from Europe, Hong Kong, India, Pakistan and Sri Lanka attended. Delegates included doctors, nurses, physiotherapists, medical students, hospital administrators and health ministry workers, representing surgery, obstetrics & gynaecology, anaesthesia, critical care, cardiology and public health (Figure 1).

Facilitator-led discussion with delegates regarding the existing delivery of care resulted in the following deficits in care being identified:

- Under-reporting of postoperative pain with no standard tool for pain assessment.
- No single route to critical care or cardiology for patients presenting to acute care services.

- Higher than expected patient-reported symptoms of anxiety and sleep disturbance post critical care, with no standardised approach to daily sedation assessment.
- Higher than expected confirmed or suspected wound infection following surgery, with no standardised assessment for surgical site infection.

Six project proposals were developed, informed by these evaluations of care, using routine clinical information captured through the NICST platforms while acknowledging limitations in the collection and use of this information [11,16,21,24]. The projects were: optimising sedation in ICU, refining referral of deteriorating patients, reducing surgical site infection after caesarean section, reducing surgical site infection after elective general surgery, improving perioperative pain assessment and improving efficiency in electrocardiogram (ECG) recording and escalation for patients presenting to hospital with symptoms of AMI. These six project proposals for the QI initiatives, alongside ethics applications (if needed), were developed. Where necessary, additional process measures were identified to enable evaluation of the impact of the QI intervention and the relevant registries are being adapted to include specific metrics.

The following provides an example of how the health systems approach was used by the collaboration to identify their priority for improvement and develop their project.

**Figure 2.**
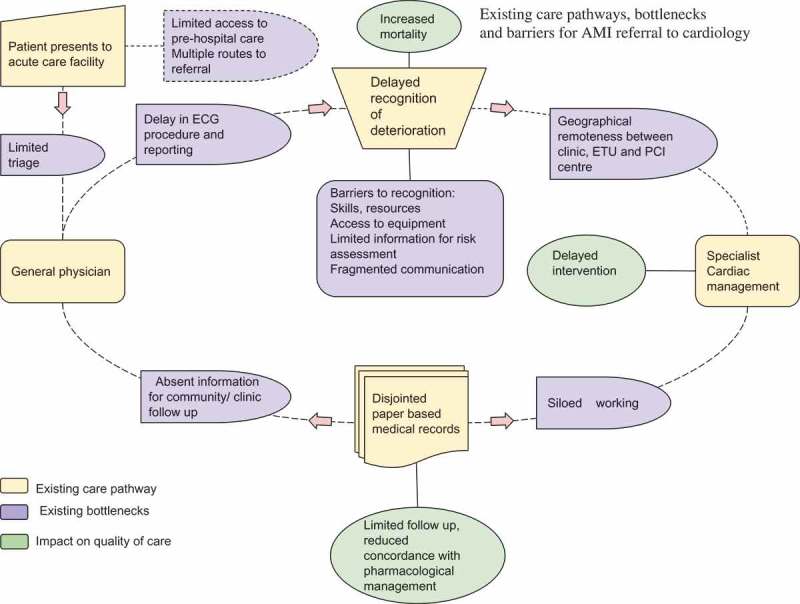
Existing care pathways, bottlenecks and barriers for AMI referrals to cardiology in Colombo, Sri Lanka.

**Figure 3.**
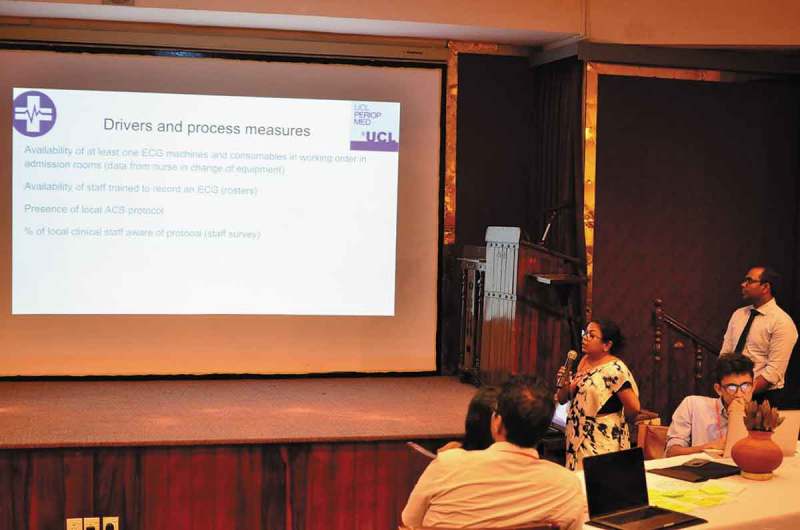
Participants sharing their project proposals with the CQIN workshop.

Indicators of care quality measured through the Sri Lankan STEMI forum and NICST’s AMI registry revealed that patients presenting with symptoms of acute coronary syndrome were experiencing delays in recognition of AMI on arrival and escalation to cardiology services [25]. Mapping of the key steps in the processes of care undertaken by delegates identified specific bottlenecks to recognition and escalation of AMI (Figure 2). These included inefficiencies in requesting, acquisition and interpretation of ECGs, and absence of a single communication route to escalate patients requiring cardiology review. Working with the faculty, the delegates then developed a proposal for a single escalation pathway for ordering an ECG and identified teams and individuals that could facilitate relocation of the ECG equipment to the admission department (Figure 3). The proposed QI initiative included leveraging the mHealth tool currently implemented as part of the existing AMI registry to help the clinical team communicate requests for care escalation. Delays in interhospital referral for patients requiring specialist intervention, also highlighted by the registry data, were considered unsuitable for a first QI project. Task shifting, role responsibilities, duplication of data entry in paper and electronic tools and challenges in changing team behaviours were elucidated as potential barriers to implementation of the QI project.

## Next steps

Delegates returning to their clinical settings will be supported to implement and evaluate their projects with resources from NICST, including support from experienced locally trained project coordinators and data collectors trained in routine surveillance. CQIN QI experts will provide ongoing support remotely through an online portal (www.nicst.com)  and using an online healthcare learning platform [22]. Reciprocal fellowships between the UK and South Asia will be utilised to support implementation of QI projects developed during the workshop and provide opportunities for shared learning. It is anticipated that evaluation of the QI projects will highlight successful and unsuccessful aspects of the solutions proposed. This learning will be invaluable to help further refinement and development of subsequent projects and will be disseminated to the wider CQIN collaboration.

## Feedback

Delegate feedback was captured with pre- and post-workshop questionnaires to assess the needs of participants and their views on how well these needs had been met. Pre-course needs analysis reported that only 25% of delegates had received formal QI training prior to the workshop. Following the workshop, 81% of delegates strongly agreed that the course had contributed to their understanding of QI and all delegates agreed that they could apply what they had learned in their own clinical settings. Two days was an acceptable time frame for 84% delegates to take leave from clinical responsibilities and 78% wished to attend subsequent activities to support implementation.

## Paper context

This short communication reports an innovative, practical approach to building capacity for interventions to improve the quality of health care in LMICs. Two fundamental aspects of successful QI are explored: how to bridge the information gap by leveraging innovative real time electronic registries, and how to engage frontline healthcare workers in data driven quality improvement using co-design and academic collaboration. Methods described here may be transferable tools for other collaborative seeking to undertake similar initiatives.

## References

[1] Meara JG, Leather AJM, Hagander L, et al Global surgery 2030: evidence and solutions for achieving health, welfare, and economic development. Lancet. 2015;386:569–6. 25924834 10.1016/S0140-6736(15)60160-X

[2] Khan MS, Hashmani FN. Political and technical barriers to improving quality of health care. Lancet. 2018;392:2146–2147. 30195399 10.1016/S0140-6736(18)32075-0

[3] Biccard BM, Madiba TE, Kluyts HL, et al Perioperative patient outcomes in the African Surgical Outcomes Study: a 7-day prospective observational cohort study. Lancet. 2018;391:1589–1598. 29306587 10.1016/S0140-6736(18)30001-1

[4] Dondorp AM, Iyer SS, Schultz MJ Critical care in resource-restricted settings. JAMA. 2016;315:753–754. 26903331 10.1001/jama.2016.0976

[5] Kruk ME, Gage AD, Joseph NT, et al Mortality due to low-quality health systems in the universal health coverage era: a systematic analysis of amenable deaths in 137 countries. Lancet. 2018;392:2203–2212. 30195398 10.1016/S0140-6736(18)31668-4 PMC6238021

[6] Ridde V Need for more and better implementation science in global health. BMJ Glob Health. 2016;1:e000115. 10.1136/bmjgh-2016-000115 PMC5321336 28588947

[7] Abimbola S The information problem in global health. BMJ Glob Health. 2016;1:e900001. 10.1136/bmjgh-2015-900001 PMC5321312 28588931

[8] WHO, World Bank Group, OECD Delivering quality health services: A global imperative for universal health coverage. NW Washington, DC; 2018:473–1000.

[9] Dare AJ, Onajin-Obembe B, Makasa EM A snapshot of surgical outcomes and needs in Africa. Lancet. 2018;391:1553–1554. 29306588 10.1016/S0140-6736(18)30002-3

[10] National Academies Crossing the Global Quality Chasm. Washington, DC: National Academies Press; 2018. 30605296

[11] Beane A, Wagstaff D, Abayadeera A, et al Surgical surveillance in resource-poor settings. Lancet. 2018;391:1571. 10.1016/S0140-6736(18)30498-7 29695338

[12] NICST [cited 2018 914]. https://nicst.com/

[13] Travis P, Bennett S, Haines A, et al Overcoming health-systems constraints to achieve the Millennium Development Goals. Lancet. 2004;364:900–906. 15351199 10.1016/S0140-6736(04)16987-0

[14] Centre for Anaesthesia and Perioperative Medicine | UCL [cited 2018 914]. https://www.ucl.ac.uk/surgery/research/tissue-and-energy/centre-anaesthesia-and-perioperative-medicine

[15] Haniffa R, Mukaka M, Munasinghe S, et al Critical care simplified prognostic model for critically ill patients in resource limited settings in South Asia. Crit Care. 2017;21:250. 29041985 10.1186/s13054-017-1843-6 PMC5645891

[16] De Silva AP, Harischandra PL, Beane A, et al A data platform to improve rabies prevention, Sri Lanka. Bull World Health Organ. 2017;95:646–651. 28867845 10.2471/BLT.16.188060 PMC5578379

[17] Rudd KE, Seymour CW, Aluisio AR, et al Association of the quick sequential (sepsis-related) organ failure assessment (qSOFA) score with excess hospital mortality in adults with suspected infection in low-and middle-income countries. JAMA. 2018;319:2202–2211. 29800114 10.1001/jama.2018.6229 PMC6134436

[18] Budrionis A, Bellika JG The learning healthcare system: where are we now? A systematic review. J Biomed Inform. 2016 12 1;64:87–92. 27693565 10.1016/j.jbi.2016.09.018

[19] Ibanez B, James S, Agewall S, et al 2017 ESC Guidelines for the management of acute myocardial infarction in patients presenting with ST-segment elevation: the Task Force for the management of acute myocardial infarction in patients presenting with ST-segment elevation of the European Society of Cardiology (ESC). Eur Heart J. 2017 8 26;39:119–177. 10.1093/eurheartj/ehx393 28886621

[20] International Surgical Outcomes Study Group Global patient outcomes after elective surgery: prospective cohort study in 27 low-, middle-and high-income countries. Br J Anaesth. 2016 11 1;117:601–609. 27799174 10.1093/bja/aew316 PMC5091334

[21] Haniffa R, De Silva A, Beane A, et al The Epimed Monitor ICU Database®: A cloud-based national critical care registry - a South Asian LMICs data platform. Rev Bras Ter Intensiva. 2018;30:251–252. 29995095 10.5935/0103-507X.20180031 PMC6031428

[22] Massive open online courses: join a collaborative online learning community and benefit from the IOE’s world-leading expertise. [cited 2018 830] http://www.ucl.ac.uk/ioe/courses/short-courses-cpd/moocs

[23] Institute for Health Improvement [cited 2018 720]. http://www.ihi.org/resources/Pages/default.aspx

[24] Hashmi M, Beane A, Memon M, et al Pakistan registry of intensive care PRICE expanding a lower middle income, clinician-designed critical care registry in South Asia. J Intensive Care Soc. 2018;1–6. 31447910 10.1177/1751143718814126 PMC6693123

[25] Schiele F, Gale CP, Bonnefoy E, et al Quality indicators for acute myocardial infarction: A position paper of the Acute Cardiovascular Care Association. Eur Heart J Acute Cardiovasc Care. 2017;6:34–59. 27574334 10.1177/2048872616643053

